# Supplementary material for: The salivary gland salivation stimulating peptide from Locusta migratoria (Lom-SG-SASP) is not a typical neuropeptide
Source: PeerJ. 2017 Jul 26;5:e3619. doi: 10.7717/peerj.3619 (PMC5533153; doi:10.7717/peerj.3619)
Supplement: Supplemental Information 1 [file peerj-05-3619-s001.pdf]

Supplementary data for :

The salivary gland salivation stimulating peptide from *Locusta migratoria* (Lom-SG-SASP) is not a neuropeptide

by Jan A. Veenstra

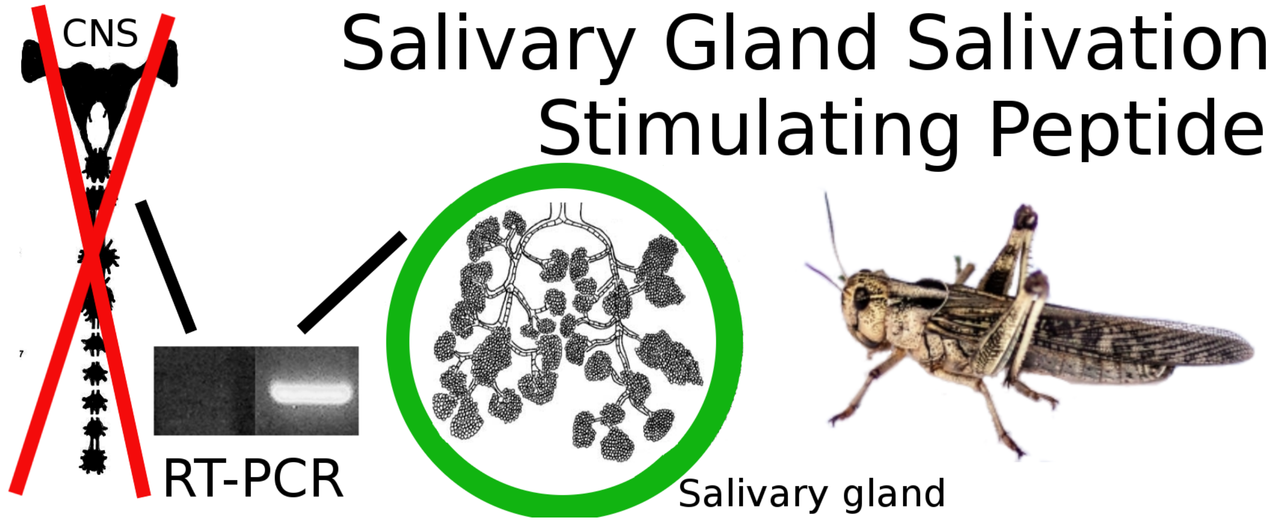

Page 2-3 **:** cDNA and protein sequences of Locusta PC1 and PC2
Page 4 : Picture of the complete gels used in figure 2 and 3 of the main manuscript
Page 5-12 : Electropherograms of the sequencing reactions of the PCR products

>*Locusta migratoria* PC1 cDNA
CGCAGGCGCCCACCGCAGCTGTTGTCTTCTGCCGCCGCTCCGCCGCCCTGCTGCCACGGAAGACGAGACCGCAGATGTCG
CTGTGGGCGCAGCTACTGGTGGCGCTGCTGGCGGGCGCCGGGCGCGCGCACGACGGCGCGCAGAACGCGCTGCACTTCAG
CAACGAGTGGGTGGTGCGGCTGGAGGGCGGACCACTGGCGGCGGCCGCGCTGGCCCGCGAGCTGGGATACCTGCTGCTGG
GCGAGGTGCCGGGCTTTCCAGACACGTACCGCATGCTGCGCTATGACCACCCGACAACGCAGAAGAGGGCGTCGCACATG
GTCACTCAGCGGCTGGCGGCCGACGCGAGGGTGCTGTGGGCAGAACAGCAGTTCTCCAAGAGCCGAGTGAAGAGGGAGGA
CCTGCCGGTGCCCAAGCAGACGCAGCTGCTCTTCAACGACGAGCTCTGGGACCAGGAGTGGTACCTGAGGGACACCCGCA
CGCGGCCGGACCTGCCGAAACTGGACCTGCACGTGCTGCCCGTGTACGCGGCGGGCATCACCGGCAAGGGCGTCCGCGTC
GTCGTGCTGGACGACGGTATCGAGAGGACGCACGAGGACCTGCGCGACAACTATGACCCGGCCATCAGCTTCGACGCCAA
CGACGACGACCCCGACCCGACGCCGCGCTACGACTTGGCGGGCTCCAACGCGCACGGCACGCGCTGCGCCGGCGAGATCG
CCATGGCCGCCAACAACGGCAAGTGCGGCGTCGGCGTCGCCTTCAACGCCCGCATCGGAGGGGTGCGCCTGCTGGATGGT
GACGTCAACGACCGCGTGGAGGGCGTGTCGCTGGGATGGGCGCACGAGCTGGTGGACATCTACAGCGTCTCCTGGGGGCC
CAACGACGACGGTCGCACCGTCGAGGGCCCGGGACGGCTCGCCACCGAGGCCATCGAGAGGGGCATCCGAGAGGGCCGCG
GCGGCAAGGGCGCCATCTACGTGTGGGCGTCGGGCAACGGCGGCAGCCGCGGCGACAACTGCAACTGCGACGGCTACATC
AACAGCGTGTACACGCTGTCCATCGGCTCGGCCTCGCAGCACGGCCAGTTCCCGTGGTACGGCGAGCGCTGCGCCGCCAC
CTTCGCCACCGCCTACTCCAGCGGCGCCTACTCCGACCAGATGATCGCGACGACGGACCTGCGCAACTCGTGCACCATCC
GGCACACGGGCACCTCCGCGGCGGCGCCTCTGGCCGCCGGCATCATCGCGCTCGCCCTGGAGGCCAACCCGGCGCTGACG
TGGCGCGACGTGCAGCACCTGGTGGCGTGGACGGCGGAGTGGGCGCCGCTGAGCGACAACCTCGGCTGGGCGCGCAACGC
GGCCGGCTTCCGCGTCAACACGCGCTTCGGCTTCGGCCTGATGAACGCCGCCGCGCTCGTCGCCGCCGCCGCCAACTGGA
CCACCGTGCCGCCCAAGGCCGTCTGCTCCACGCCCGTGGCGCCCACGGCCAACACGTCGATAAAGCCAGGCGTGCTGACG
GTGGCGCACTTCCACACGGACGGCTGCGCCGGCACCGAGCGAGAAGTCAACTTCCTGGAGCACGTGGAGCTGCAGGTGAA
CCTGCGCTACTCGCTGCGGGGCGCCCTCGAGATACACCTCACGTCTGCTGCAGGAACGAAGGTGGAGTTGCTGAGTCCAA
GGAAGCTGGATAAATCCAGTGAAGGTTTCAAAGACTGGAAATTCATGTCAGTTCTAACGTGGGGAGAGAGACCAGCTGGC
ATTTGGACAGTAGAAATCATCGACAGGCAAGGAGGCTCCACGAACCGCACCGGGGAGCTGGTGTCGGCGTCGCTGATCCT
GCACGGCACCAAGGAGGCGCCGCACCACCTGAGGAGCGGGCCCAGGGCCTACGACGACGACTACAACCACGTGCACAAGA
AGATGGCGAACGCTGAGCCCATGGAGAAGCGGCTACCGCTGGATGTTTTGCAAGAGACGGACGCTGCGCTGGACTGGGCG
GACATCATCGGTATCCCACTATCGTTAAGGAGCCAGCCTGATTTCAGGAGTATGCTGCTGAAGCTGTGGCTTAGTCGCAT
TCAAGAAAAGCAGGCAACATAACAACCTCAAGCCTGCAACACACTGGAGGAACAGACTGTACAGTACAATAAGCGACACT
GTTTGCCATATAGTTGTATTACTGAAAGCATAAATAAATAATAGGTAATAAAGTGCTATTTTAATAAAAAA
>*Locusta migratoria* PC1 protein
MSLWAQLLVALLAGAGRAHDGAQNALHFSNEWVVRLEGGPLAAAALARELGYLLLGEVPGFPDTYRMLRYDHPTTQKRAS
HMVTQRLAADARVLWAEQQFSKSRVKREDLPVPKQTQLLFNDELWDQEWYLRDTRTRPDLPKLDLHVLPVYAAGITGKGV
RVVVLDDGIERTHEDLRDNYDPAISFDANDDDPDPTPRYDLAGSNAHGTRCAGEIAMAANNGKCGVGVAFNARIGGVRLL
DGDVNDRVEGVSLGWAHELVDIYSVSWGPNDDGRTVEGPGRLATEAIERGIREGRGGKGAIYVWASGNGGSRGDNCNCDG
YINSVYTLSIGSASQHGQFPWYGERCAATFATAYSSGAYSDQMIATTDLRNSCTIRHTGTSAAAPLAAGIIALALEANPA
LTWRDVQHLVAWTAEWAPLSDNLGWARNAAGFRVNTRFGFGLMNAAALVAAAANWTTVPPKAVCSTPVAPTANTSIKPGV
LTVAHFHTDGCAGTEREVNFLEHVELQVNLRYSLRGALEIHLTSAAGTKVELLSPRKLDKSSEGFKDWKFMSVLTWGERP
AGIWTVEIIDRQGGSTNRTGELVSASLILHGTKEAPHHLRSGPRAYDDDYNHVHKKMANAEPMEKRLPLDVLQETDAALD
WADIIGIPLSLRSQPDFRSMLLKLWLSRIQEKQAT-
>*Locusta migratoria* PC2 cDNA (incomplete)
CTGGGGGCGGCGAGCGCACTGCACCTGGTCCTCGCCCTGGGGCTGGCCGCCGCCGCCGCCGCCGCCACCGCCGCGGC
CGGCAGGCCTCGCGAGGTCTTCACCAACTCGTTCCTGGTGCGGCTGCGGGGCGCGCCCGGCGCCGCCGTCGCAGACCAGG
TGGCCGCGCGCACCGGCTTCCTCAACCTGGGACCGGTGCTGGGCTCGGACCGCGAGTTCCACTTCGTGCACCGCGCGCTG
CCGCACGCCCGCAGCAAGCGCAGCATCCCGCACATGCGGCGCCTCAAGGTGGACCCGCTGGTGGAGGCGGCGGTGCAGCA
GACGGGCTTCAGACGGGTGAAGCGCGGCTACAAGCCGCTGCGCGTCGAGAACCTGGTGAAGAACATGCGGCCGCAGCGCG
ACCCCGCAGACCCCTACTTCCCGTTCCAGTGGTACCTCAAGAACACCGGCCAGAACGGCGGCAAGGCCAAGCTCGACCTC
AACGTCGAGGCCGCCTGGGCGCAGGGCGTCACCGGCAAGAACGTCACCACCGCCATCATGGACGACGGTGTGGACTACAT
GCACCCAGATCTCAAACATAATTATAACGCGCGCGCCAGCTACGACTTCAGCAGCAACGACCCGTACCCCTACCCGCGCT
ACACCGACGACTGGTTCAACAGCCACGGGACGCGGTGCGCCGGAGAGGTGGCAGCCGCCCGGGACAACGGCGTGTGCGGC
GTGGGCGTCGCCTACGACTCCAAGATAGCAGGCATCCGCATGCTGGATCAGCCGTACATGACGGACCTGATCGAGGCCAA
CTCGATGGGCCACGAGCCCAACCTTATCGACATCTACAGCGCCTCCTGGGGGCCCACCGACGACGGCAAGACGGTGGACG
GGCCGCGCAACGCCACCATGCGCGCCATCGTCCGCGGCGTCAACGAGGGCAGGAACGGGCTGGGCAACATCTACGTGTGG
GCGAGCGGCGACGGCGGCGAGGACGACGACTGCAACTGCGACGGCTACGCGGCCAGCATGTGGACCGTGTCCATCAACAG
CGCCATCAACGACGGCCAGAACGCGCACTACGACGAGAGCTGCTCCTCCACGCTCGCCTCCACCTTCAGCAACGGCGCCA
AGGACCCCAACACCGGAGTCGCGACGACGGACCTGTACGGCAAGTGCACCACCACGCACTCGGGCACGTCGGCCGCCGCT
CCGGAGGCGGCGGGAGTCTTCGCGCTGGCGCTGGAGGCCAACCCGCAGCTGACGTGGCGCGACATCCAGCACCTGACGGT
GCTCACGTCGAAGCGCAACTCGCTGTTCGACGCGAAGCGGCGCTTCCACTGGACGATGAACGGCGTGGGGCTGGAGTTCA
ACCACCTGTTCGGCTTCGGCGTGCTGGACGCGGGCGCCATGGTGGCGCTGGCCAAGCAGTGGCGCACCGTGCCCGCCAGG
TTCCACTGCGAGGGCGGCTCCCTCAACGAACCGCATGAGATCCCGTCGCGCGGGCCGCTGGTGCTGAAGATCTCGACGGA
CGCGTGCCGCGGCCTGGACACGCAGGTGGACTACCTGGAACACGTGCAGGCCGTCATCACGCTCAACGCGACGCGACGTG
GTGACCTCGAGCTCTTCCTCACCTCGCCCATGGGGACCAGATCGATGATCTTGAGCAGACGTGTTAACGATGATGATCAC
CGTGATGGCTTCACCAAATGGCCATTCATGACAACTCACACCTGGGGAGAATATCCACAGGGTACATGGACACTGGAGGT

TGGCTTCAATTCACCAATACCACAATCAGGCTTCTTAAAGGAATGGACTCTAATGTTGCATGGCACACGGGACCCACCAT
ATAGTGAGCTACCTGTCACAGACCCACACTCCAAGTTAGCCATCGTGAAGAAGGCCCATGAGGAGCGCAGCAAGTTGTAG
GACATCCAAAAGCTGTGACCTTATATTGTAATAGCTGTTAGACATGGTATGCCACTTTGATTCATTGGCTAAACCCACTA
ACCTACAGTTTTTCAGTAACACAATCAACTCATTCTGAGTTTTTTGTAGTAGCTTCCCTACTTTGGTACAATTATTTTAA
TTAGTAAGTATCATATCCTGTTTTGTCCTTTATGACATACCACCAAGTTTCAAAGGTCTTACCATGTCTAGGACCCTCAT
AGAGCAGATTCCTGGAAATACTTATAAAACTTCTTCATCCATAATTTCCTTCTGAGCTCTGCTGTGTTGGTATCAATATT
ATTTGAGTGATTCAGGTGTTCAACAATAGATGAACCTTTTACTTAATGTTCAGTATTGTGCAGGTTTTAAAATAAAAGCA
ACACAGCAGCTGTAACTCTTTTTCTAAAAGCAAAGAAGAAAAGCTTCTTTCATACAGCAACCTTCCATGTAGAGAGAACA
AATTTGAGGTAGAGAGTGCAAACCAATATGAAAGTTAATATATTTCTGTATAATACTACAAACTAAAGATCTGCATCAAA
ATTATTGGTATTGGATGTATAACAAAGTAATGCCGAGATAAAATTACTTATTATATTCTGTTAATAGCTGTATGAACAAA
ATGAATGGGGAAAAGGGTG
>*Locusta migratoria* PC2 protein (incomplete N-terminal)
....LGAASALHLVLALGLAAAAAAAATAAAGRPREVFTNSFLVRLRGAPGAAVADQVAARTGFLNLGPVLGSDREFHFV
HRALPHARSKRSIPHMRRLKVDPLVEAAVQQTGFRRVKRGYKPLRVENLVKNMRPQRDPADPYFPFQWYLKNTGQNGGKA
KLDLNVEAAWAQGVTGKNVTTAIMDDGVDYMHPDLKHNYNARASYDFSSNDPYPYPRYTDDWFNSHGTRCAGEVAAARDN
GVCGVGVAYDSKIAGIRMLDQPYMTDLIEANSMGHEPNLIDIYSASWGPTDDGKTVDGPRNATMRAIVRGVNEGRNGLGN
IYVWASGDGGEDDDCNCDGYAASMWTVSINSAINDGQNAHYDESCSSTLASTFSNGAKDPNTGVATTDLYGKCTTTHSGT
SAAAPEAAGVFALALEANPQLTWRDIQHLTVLTSKRNSLFDAKRRFHWTMNGVGLEFNHLFGFGVLDAGAMVALAKQWRT
VPARFHCEGGSLNEPHEIPSRGPLVLKISTDACRGLDTQVDYLEHVQAVITLNATRRGDLELFLTSPMGTRSMILSRRVN
DDDHRDGFTKWPFMTTHTWGEYPQGTWTLEVGFNSPIPQSGFLKEWTLMLHGTRDPPYSELPVTDPHSKLAIVKKAHEER
SKL-

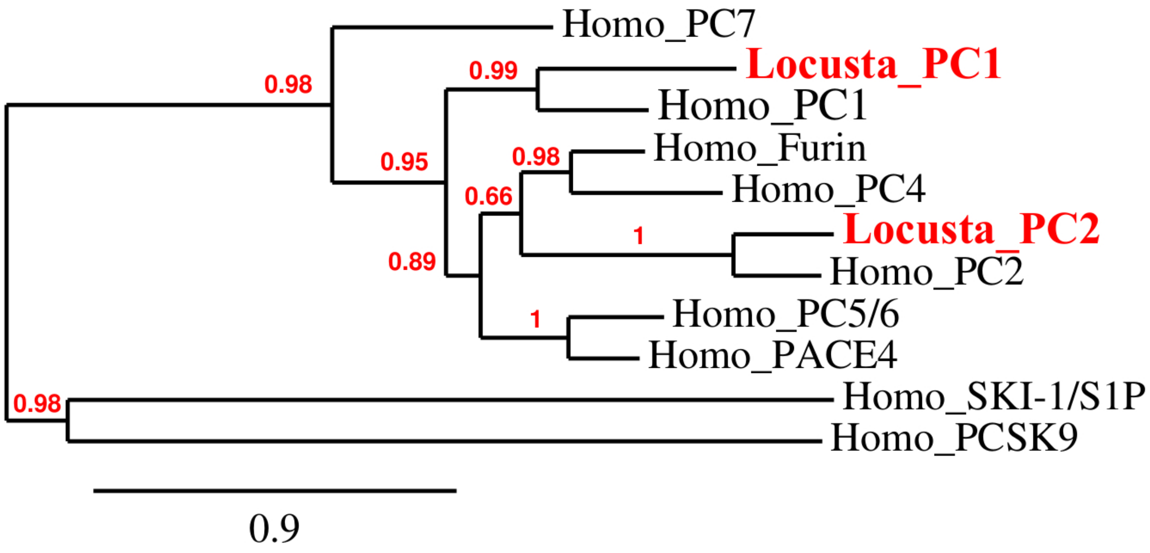

Phylogenetic tree of two *Locusta* convertases and the human members and the of the kex-like protease family, illustrating that the latter are orthologs of vertebrate PC1 and PC2.

Pictures of the gel cropped in figs 2 and 3.

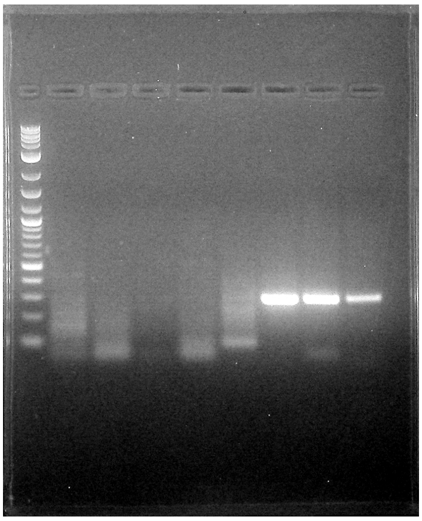

Fig. 2. SG-SAGP

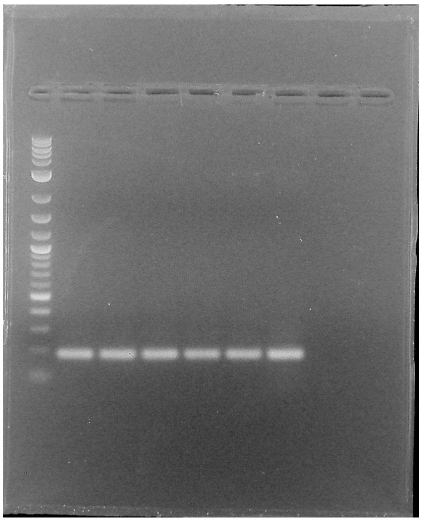

Fig. 2 actin

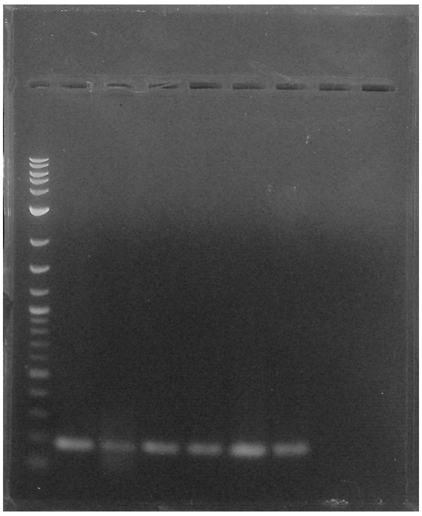

Fig. 3. PC1

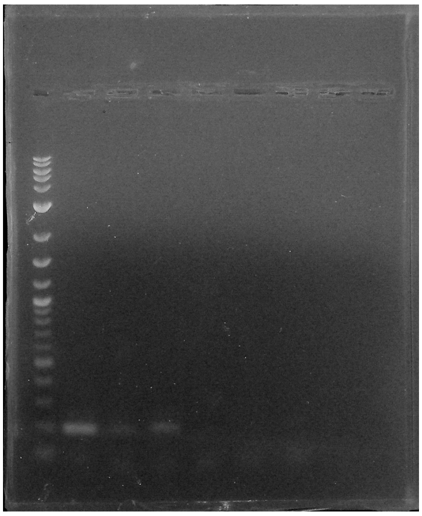

Fig. 3. PC2

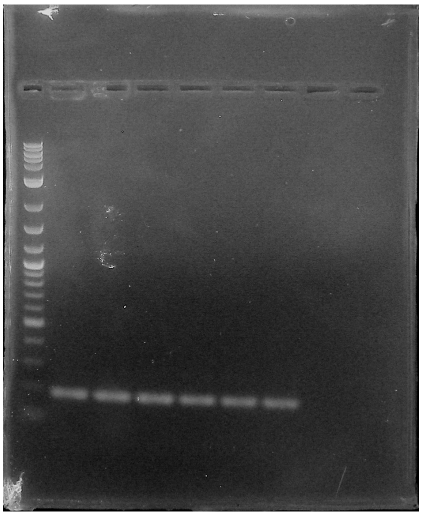

Fig. 3. actin

All gels 1.5% TAE, first lane markers (0.1, 0.2, 0.3, 0.4, **0.5**, 0.6, 0.7, 0.8, 0.9, **1.0**, 1.2, 1.5, 2.0, **3.0**, 4.0, 5.0, 6.0, 8.0 and 10.0 kilo base pairs). Expected sizes of the PCR products for SG-SASP, PC1, PC2 and actin cDNAs respectively 298, 181, 195 and 189 base pairs.

PCR product of SG-SASP forward primer

Applied Biosystems

KB 1.4.1.8 KB.bcp

28418543

KB_3730_POP7_BDTv3.mob

Signal: G:1204 A:1843 T:1989 C:2507 AvgSig: 1885 C#:78 W:D19 Plate Name:AA1000045334 TS:45 CRL:237 QV20+:242

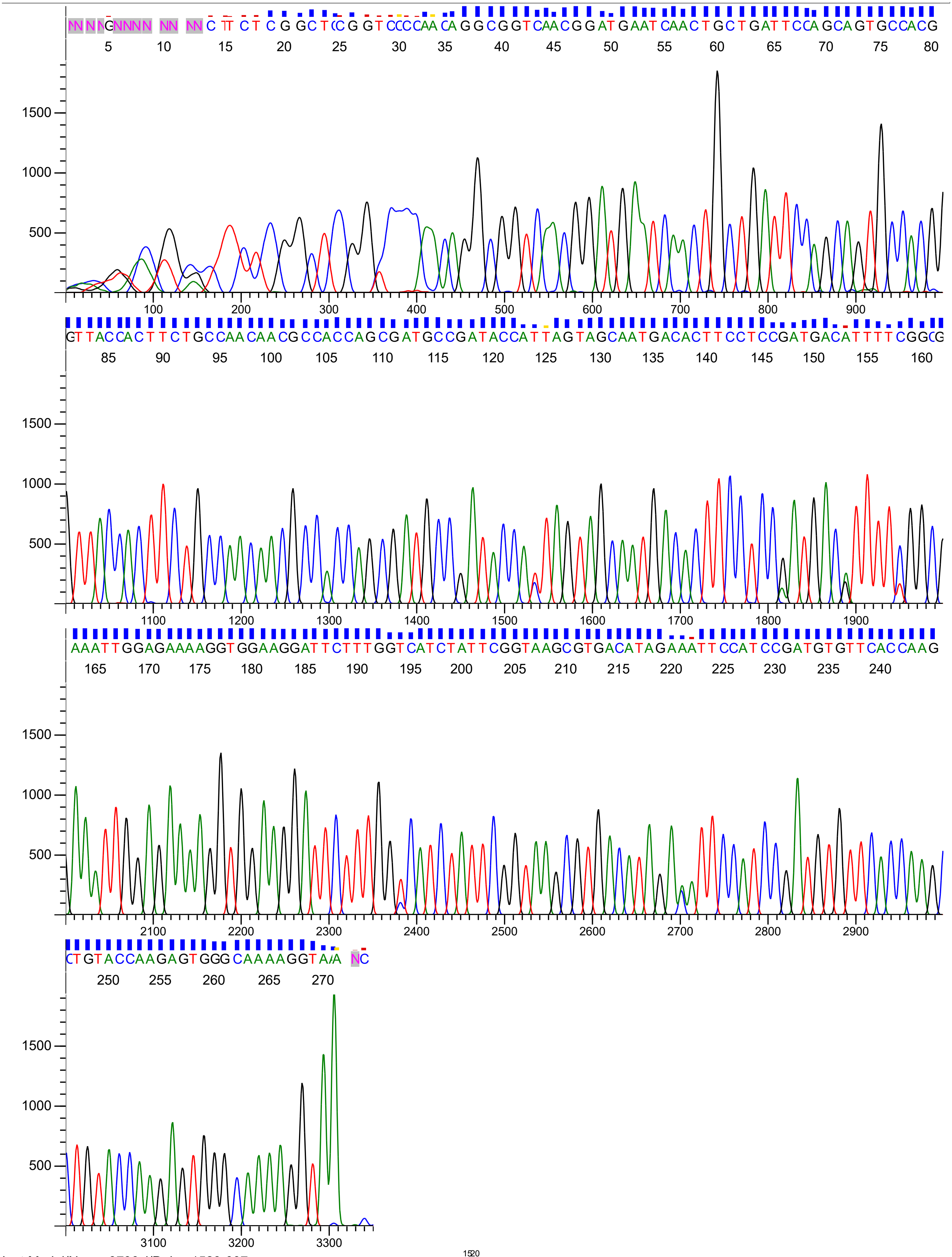

Inst Model/Name:3730xl/Daisy-1523-007

Sequence Scanner Software 2 v2.0

Pure Base QVs: 15 20

Mixed Base QVs: 10 16

PCR product of SG-SASP reverse primer

Applied Biosystems

KB 1.4.1.8 KB.bcp

28426124

KB_3730_POP7_BDTv3.mob

Signal: G:4259 A:6026 T:7357 C:8853 AvgSig: 6623 C#:80 W:B19 Plate Name:AA1000045305 TS:48 CRL:242 QV20+:229

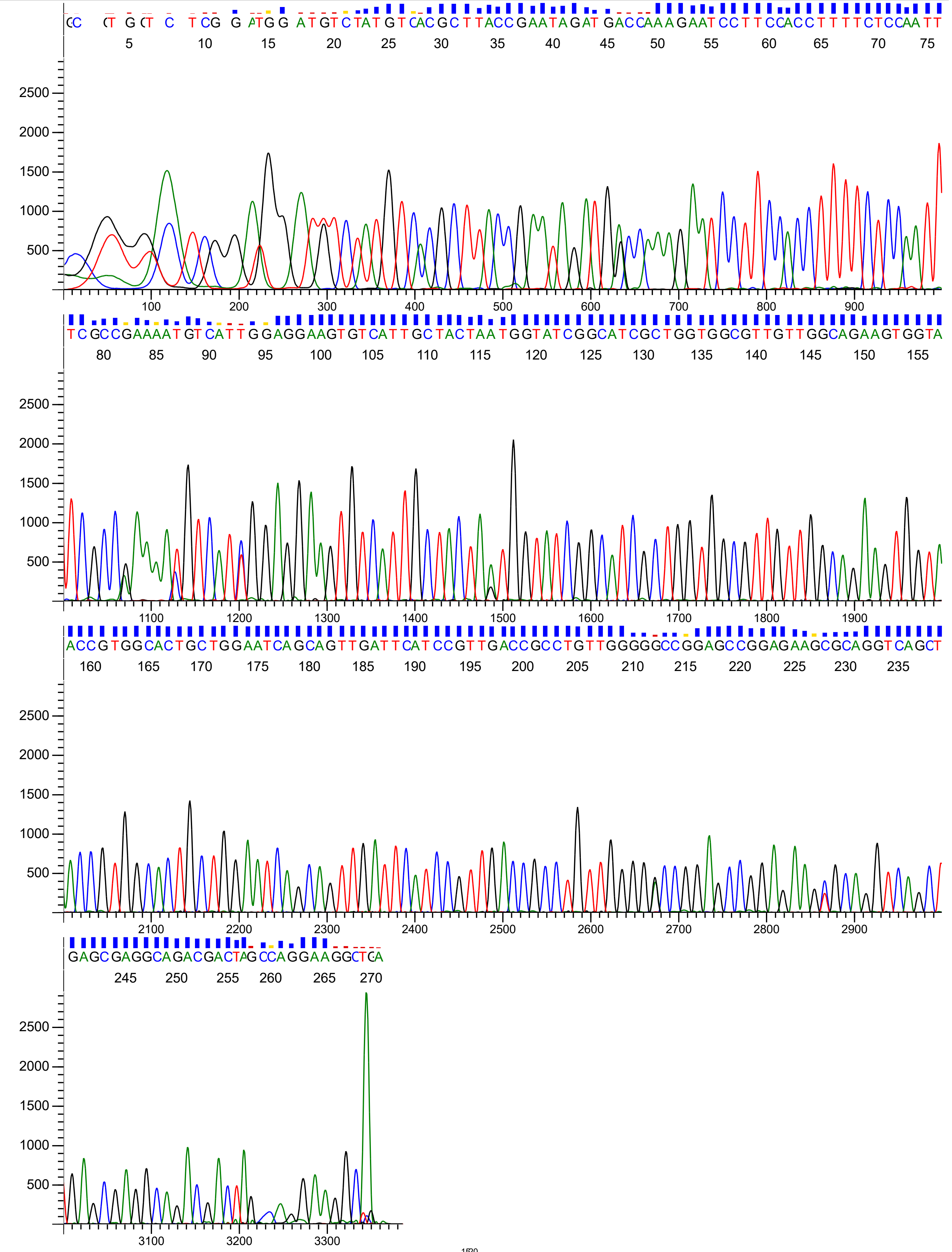

Inst Model/Name:3730xl/Colonia-28124-002

Sequence Scanner Software 2 v2.0

Pure Base QVs: 15 20

Mixed Base QVs: 10 16

PCR product of PC1 forward primer

Applied Biosystems

KB 1.4.1.8 KB.bcp

29392100

KB_3730_POP7_BDTv3.mob

Signal: G:6188 A:5951 T:6719 C:8813 AvgSig: 6917 C#:94 W:C23 Plate Name:AA1000048285 TS:37 CRL:124 QV20+:130

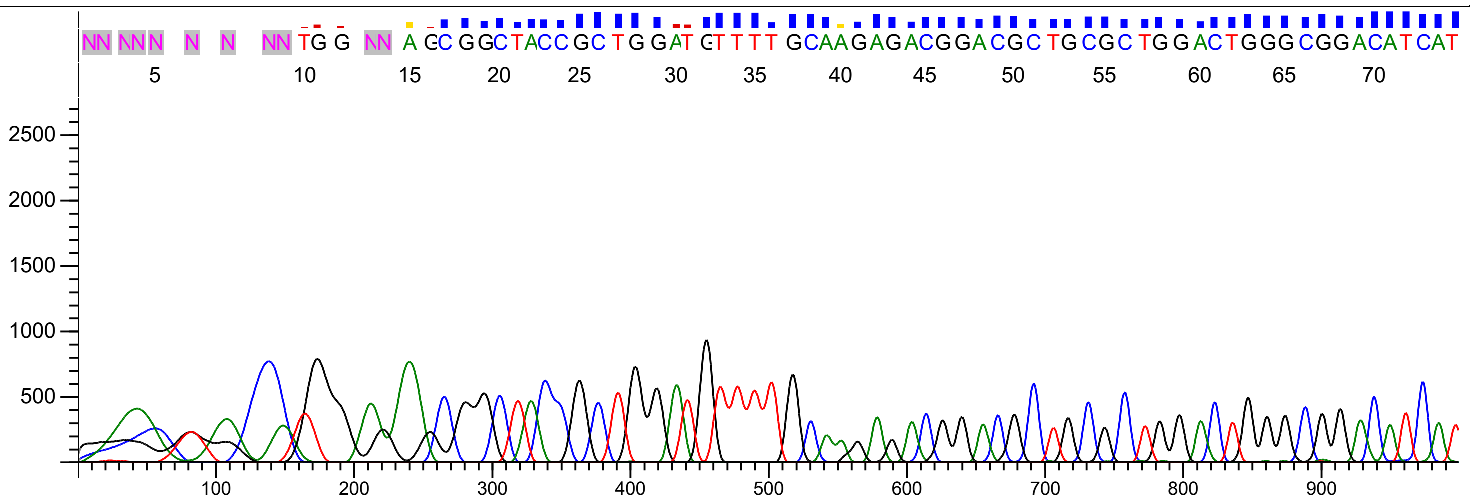

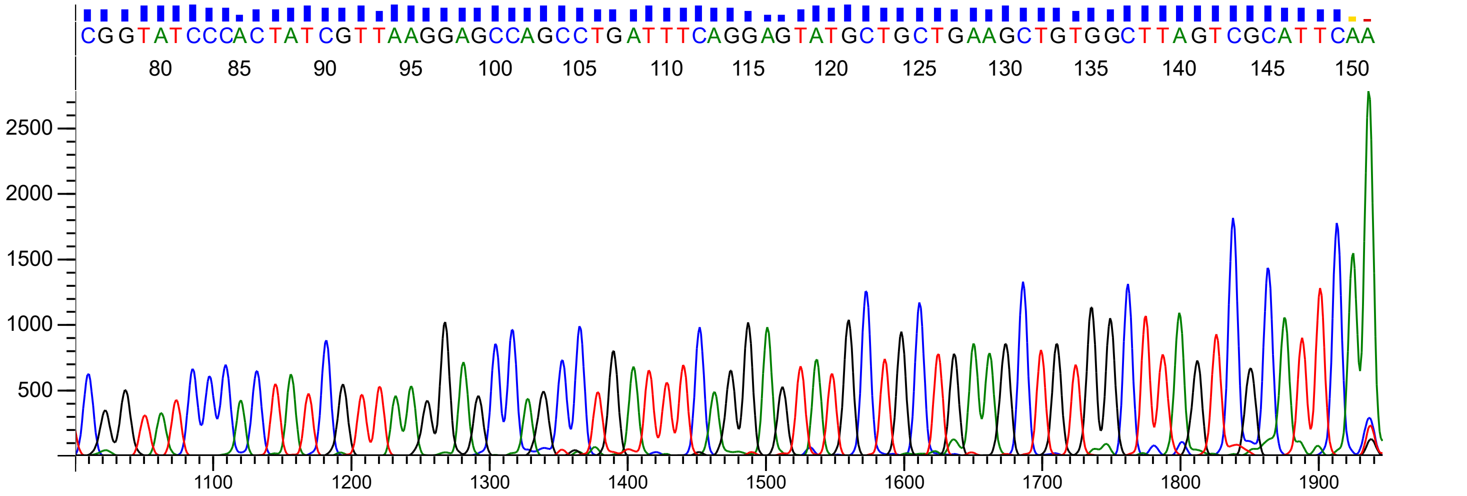

Inst Model/Name:3730xl/Lucy-18127-012

Sequence Scanner Software 2 v2.0

Pure Base QVs: 15 20

Mixed Base QVs: 10 16

PCR product of PC1 reverse primer

Applied Biosystems

KB 1.4.1.8 KB.bcp

29397629

KB_3730_POP7_BDTv3.mob

Signal: G:3794 A:2655 T:4435 C:6608 AvgSig: 4373 C#:17 W:P5 Plate Name:AA1000048249 TS:40 CRL:132 QV20+:138

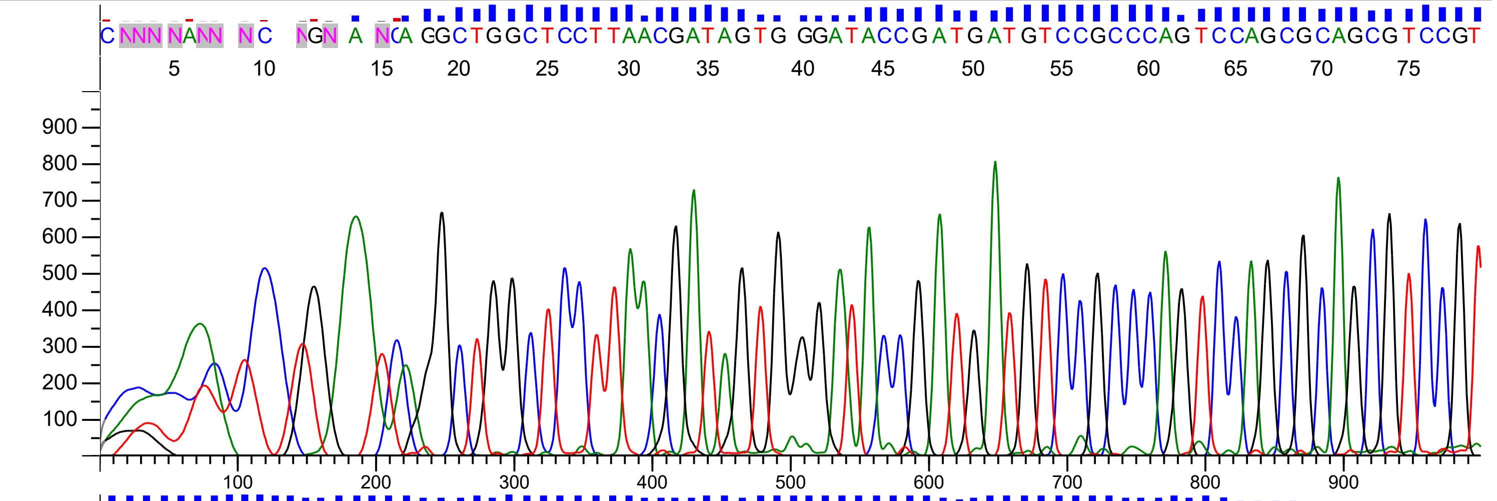

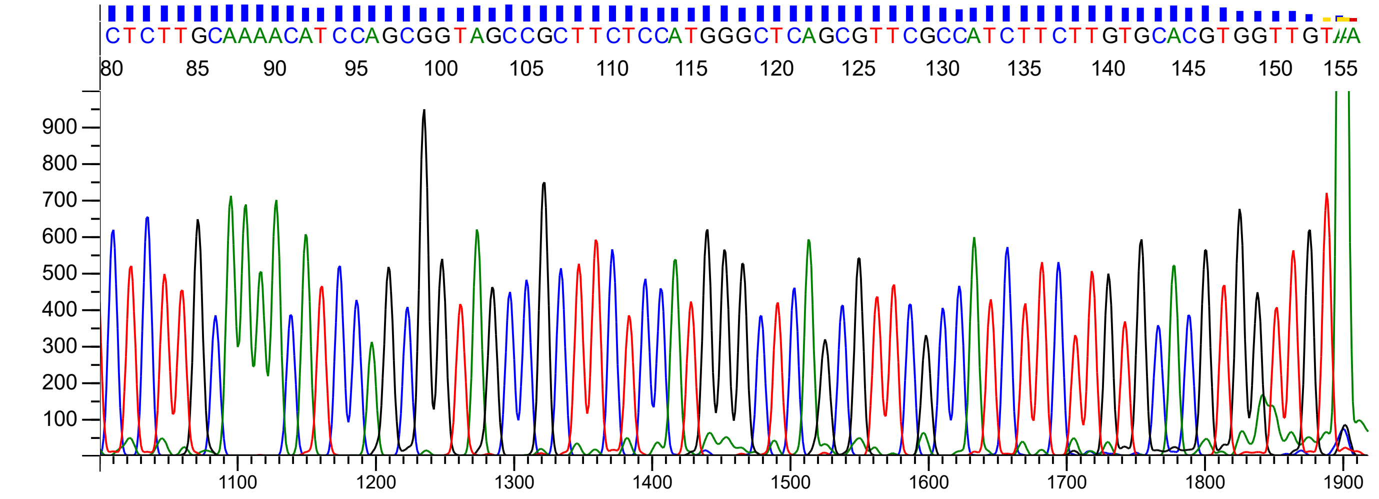

Inst Model/Name:3730xl/Daisy-1523-007

Sequence Scanner Software 2 v2.0

Pure Base QVs: 15 20

Mixed Base QVs: 10 16

PCR product of PC2 forward primer

Applied Biosystems

KB 1.4.1.8 KB.bcp

29397692

KB_3730_POP7_BDTv3.mob

Signal: G:2133 A:2819 T:2739 C:3935 AvgSig: 2906 C#:40 W:J11 Plate Name:AA1000048249 TS:51 CRL:140 QV20+:147

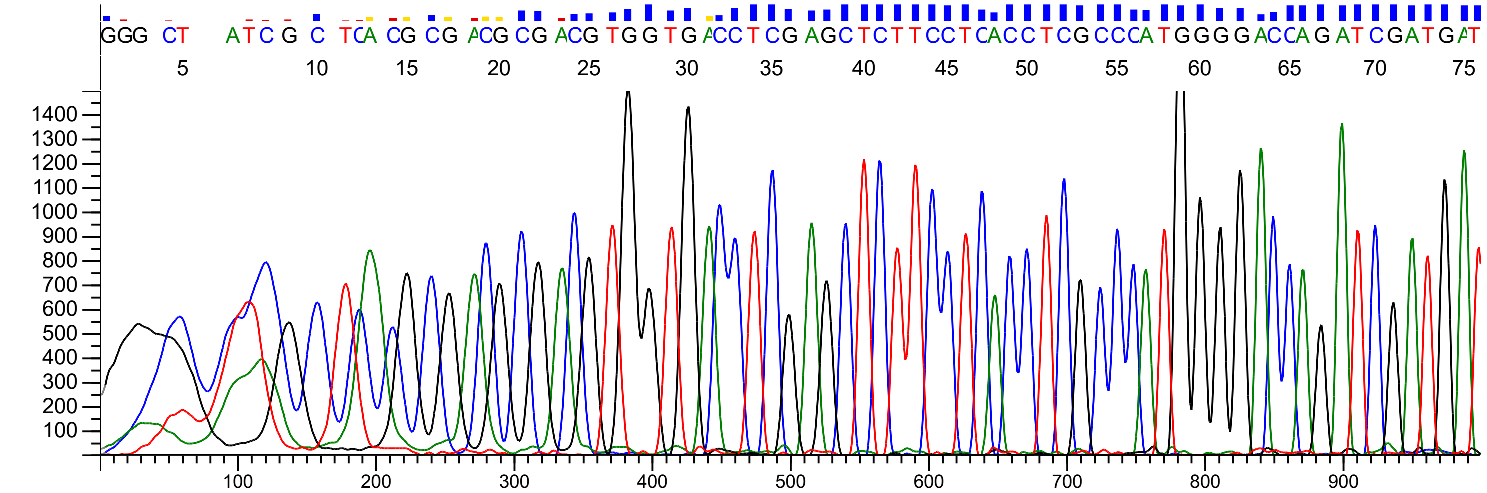

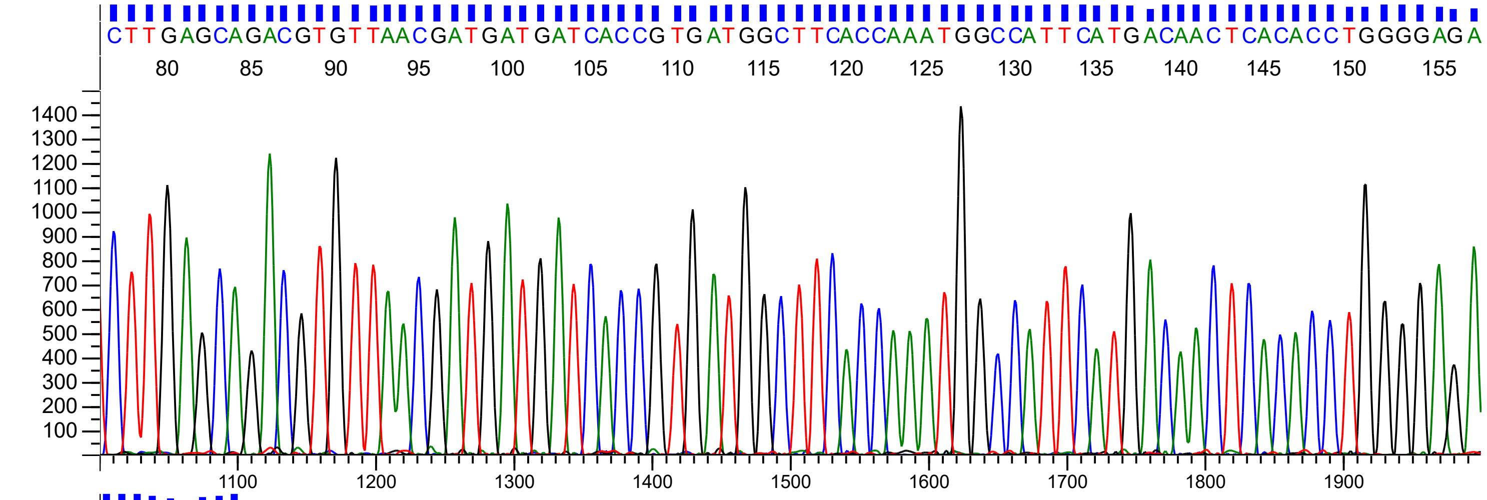

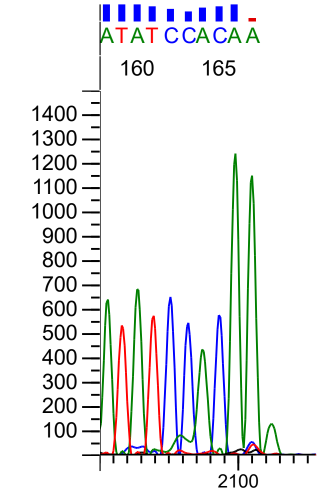

Inst Model/Name:3730xl/Daisy-1523-007

Sequence Scanner Software 2 v2.0

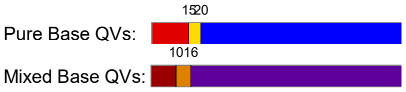

PCR product of PC2 reverse primer

Signal: G:3507 A:3315 T:4442 C:5806 AvgSig: 4267 C#:79 W:B17 Plate Name:AA1000048249 TS:41 CRL:137 QV20+:146

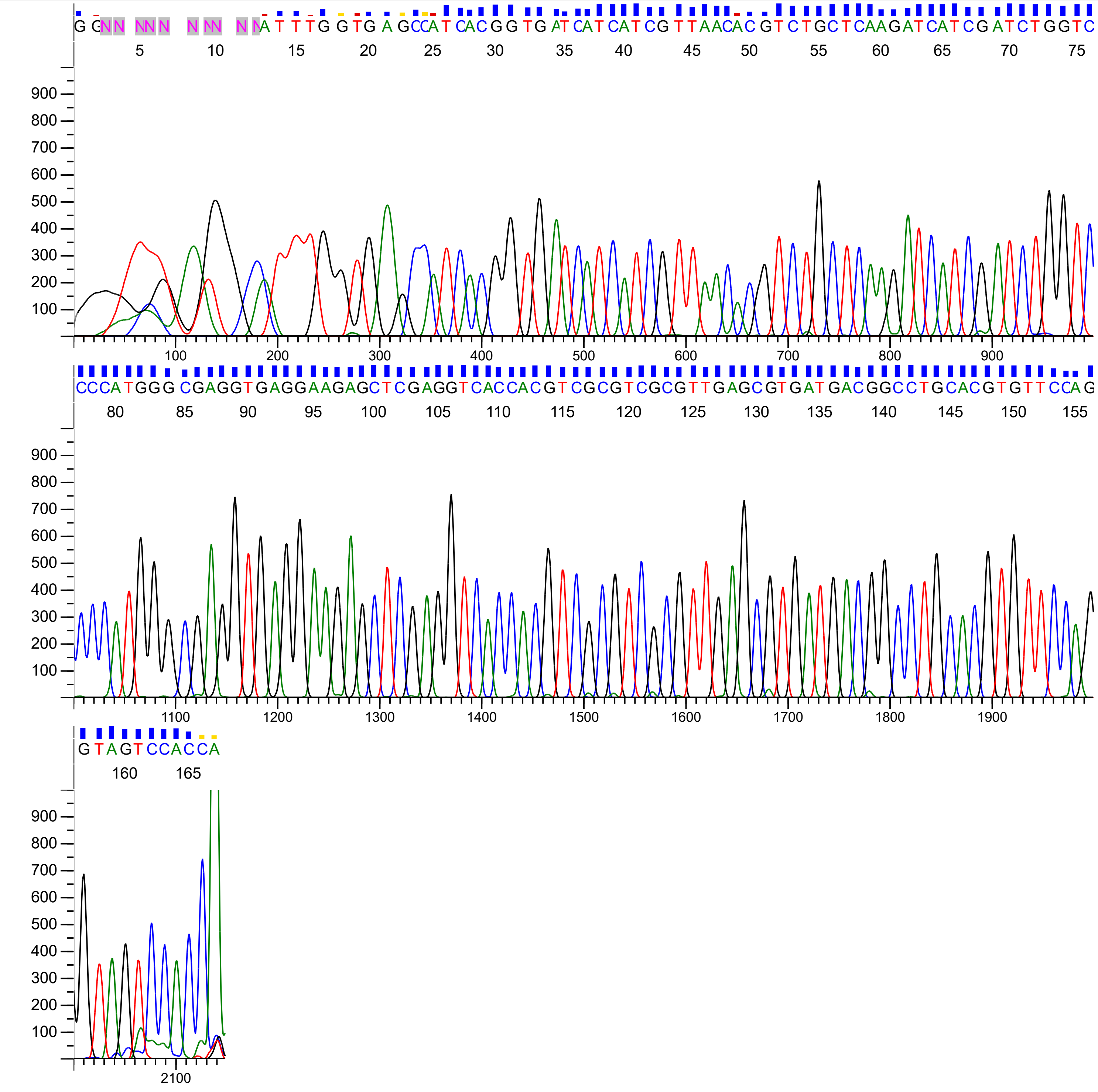

Inst Model/Name:3730xl/Daisy-1523-007

Sequence Scanner Software 2 v2.0

Pure Base QVs: 15 20

Mixed Base QVs: 10 16

PCR product of actin forward primer

Applied Biosystems

KB 1.4.1.8 KB.bcp

29397890

KB_3730_POP7_BDTv3.mob

Signal: G:2792 A:2843 T:3121 C:2588 AvgSig: 2836 C#:31 W:B5 Plate Name:AA1000048248 TS:51 CRL:135 QV20+:138

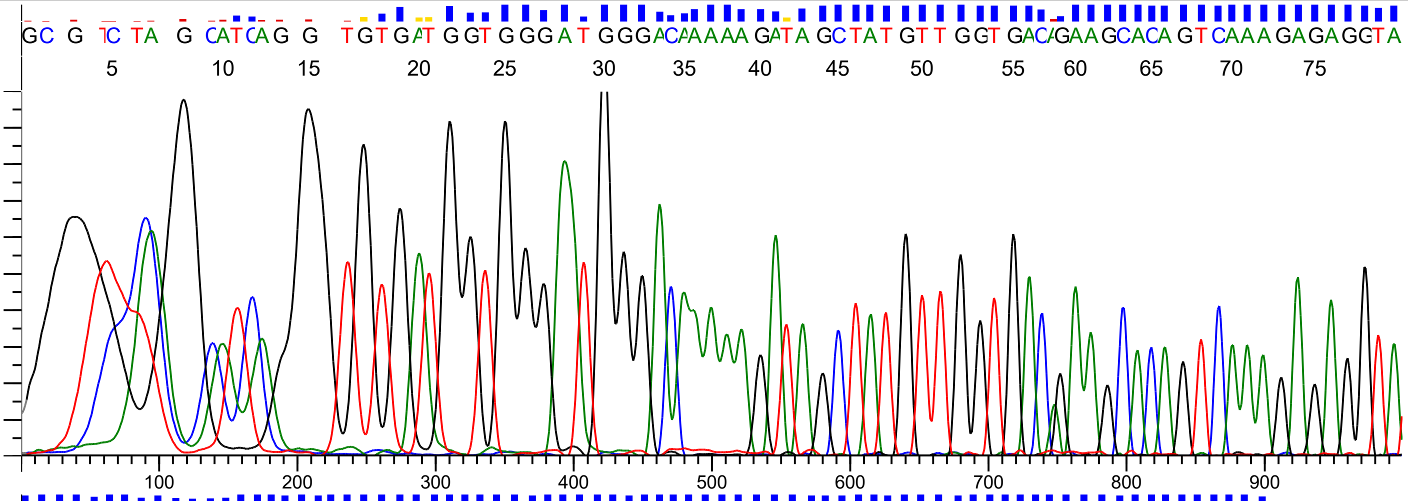

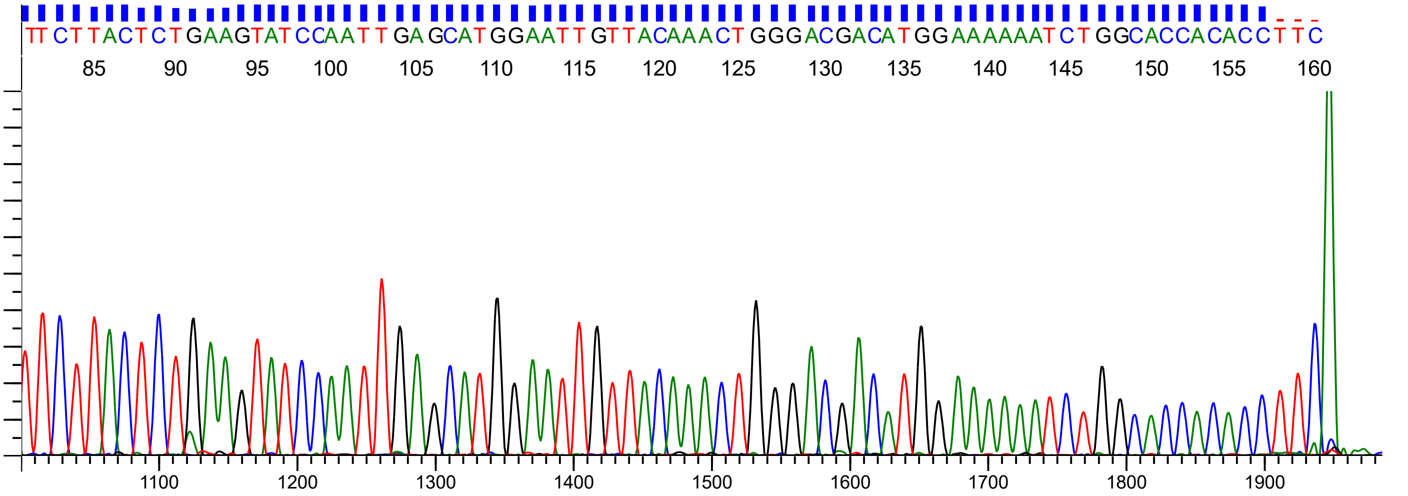

Inst Model/Name:3730xl/Merlin-1521-038

Sequence Scanner Software 2 v2.0

Pure Base QVs:

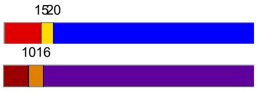

Mixed Base QVs:

PCR product of actin reverse primer

Applied Biosystems

KB 1.4.1.8 KB.bcp

29397827

KB_3730_POP7_BDTv3.mob

Signal: G:2787 A:4291 T:6526 C:6623 AvgSig: 5056 C#:92 W:F23 Plate Name:AA1000048249 TS:41 CRL:126 QV20+:133

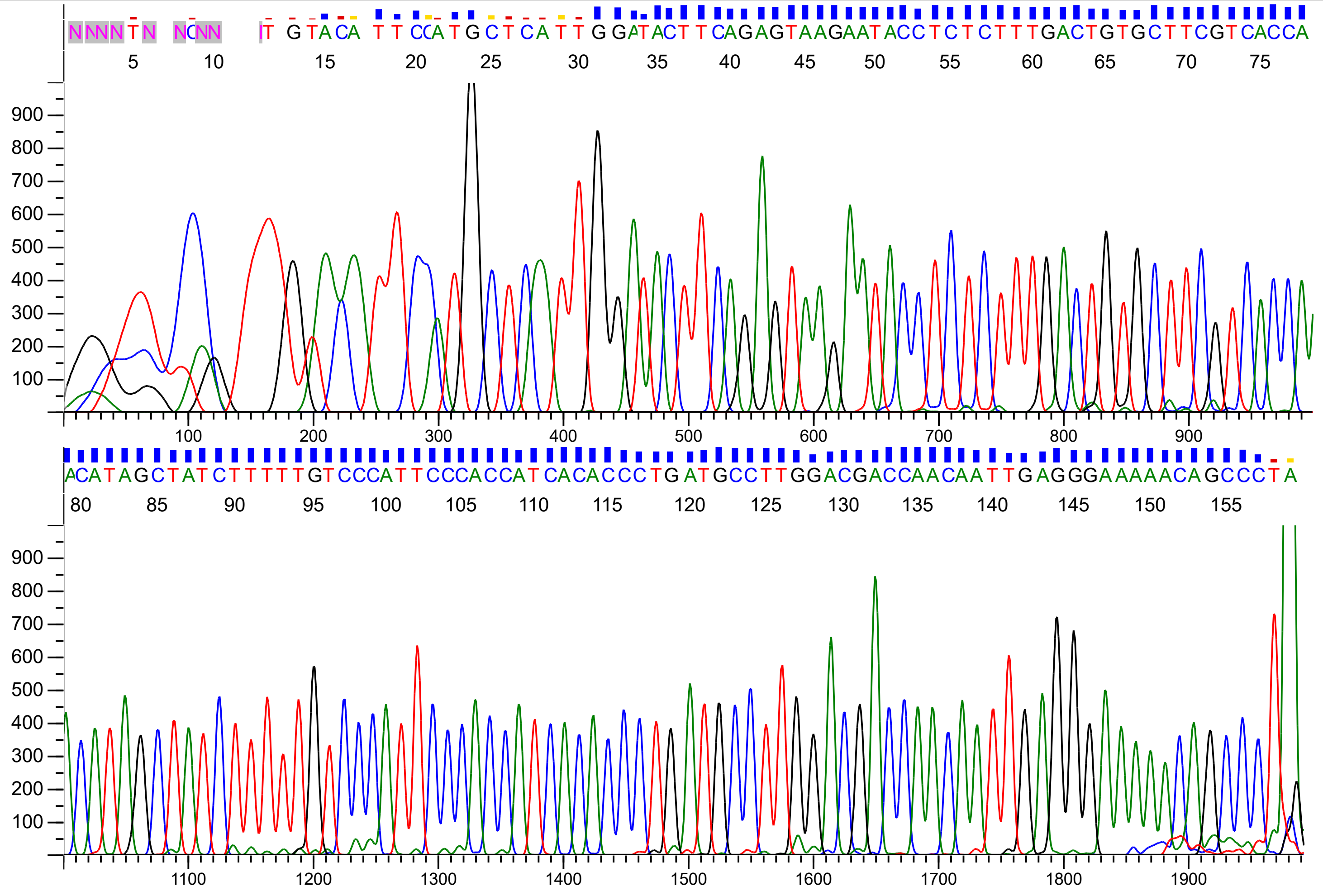

Inst Model/Name:3730xl/Daisy-1523-007

Sequence Scanner Software 2 v2.0

Pure Base QVs: 15 20

Mixed Base QVs: 10 16
